# The Flagellum Attachment Zone: ‘The Cellular Ruler’ of Trypanosome Morphology

**DOI:** 10.1016/j.pt.2015.12.010

**Published:** 2016-04

**Authors:** Jack D. Sunter, Keith Gull

**Affiliations:** 1 Sir William Dunn School of Pathology, University of Oxford, Oxford OX1 3RE, UK

**Keywords:** *Trypanosoma brucei*, flagellum, FAZ, morphology, cytoskeleton

## Abstract

A defining feature of *Trypanosoma brucei* cell shape is the lateral attachment of the flagellum to the cell body, mediated by the flagellum attachment zone (FAZ). The FAZ is a complex cytoskeletal structure that connects the flagellum skeleton through two membranes to the cytoskeleton. The FAZ acts as a ‘cellular ruler’ of morphology by regulating cell length and organelle position and is therefore critical for both cell division and life cycle differentiations. Here we provide an overview of the advances in our understanding of the composition, assembly, and function of the FAZ.

## Trends

The flagellum attachment zone (FAZ) is a large cytoskeletal structure that connects the flagellum skeleton to the cell body cytoskeleton through the membrane of both the flagellum and the cell body. The structure can be divided into eight zones.

The FAZ is a key morphogenetic structure regulating both cell length and organelle positioning.

Recent studies have identified numerous FAZ proteins. The function of a subset of these proteins has been studied by RNAi, revealing a range of different phenotypes from flagellum detachment to organelle positioning effects.

The assembly of the FAZ occurs at its proximal end – the opposite polarity to that of the flagellar axoneme and paraflagellar rod.

## FAZ: A Key Regulator of Morphogenesis in Trypanosomes

*Trypanosoma brucei* is a protozoan parasite that causes human African trypanosomiasis and nagana in cattle. The *T. brucei* trypomastigote is long and slender with a single flagellum that emerges near the posterior of the cell and is subsequently attached to the cell body for most of its length as it follows a defined helical path towards the anterior of the cell. A complex structure termed the FAZ mediates this lateral attachment 1, 2, 3. The FAZ is a key regulator of cell morphogenesis; disruption of FAZ assembly has been linked to morphogenetic changes such as flagellum detachment and alterations in cell shape and size 4, 5, 6, 7, 8, 9, 10.

**Figure 1:**
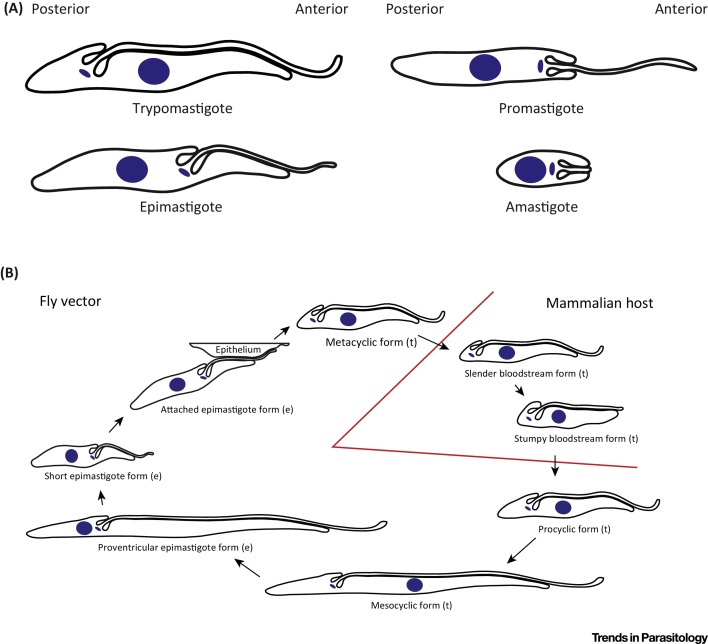
The Morphologies of Kinetoplastid Parasites and the Life Cycle of *Trypanosoma brucei*. (A) Cartoons showing the common morphologies of kinetoplastid parasites with the anterior and posterior of the cell indicated. The nucleus is the large blue circle and the kinetoplast (mitochondrial DNA) is the small blue oval located at the base of the flagellum. The trypomastigote morphology has the kinetoplast posterior to the nucleus with a long attached flagellum. In the epimastigote and promastigote, the kinetoplast is anterior to the nucleus, but whereas the epimastigote has a long attached flagellum, in the promastigote the flagellum emerges from the anterior pole of the cell. In the amastigote, the kinetoplast is anterior to the nucleus with a small cell body and the flagellum does not emerge beyond the cell body. (B) A simplified cartoon showing the life cycle of *T. brucei*. The metacyclic form infects the mammalian host where it differentiates first into the slender bloodstream form followed by the stumpy bloodstream form. The fly vector then takes up the stumpy bloodstream form where it differentiates through several stages before differentiating into the mammalian infective metacyclic form in the salivary gland of the fly. For most of its life cycle *T. brucei* exists as a trypomastigote [indicated by (t)] except for the proventricular epimastigote, the short epimastigote, and the attached epimastigote forms, where it has epimastigote morphology [indicated by (e)].

The early classifications of trypanosomes relied on their morphological characteristics, with the terminology for trypanosome shape defined by the relative position of the kinetoplast and the nucleus coupled with the length of attached flagellum (Figure 1A) [11]. During the *T. brucei* life cycle there are substantial changes in cell morphology that appear adapted for pathogenicity and proliferation in both the mammalian host and the insect vector; these changes have been linked to modulations of the protein composition of the FAZ (Figure 1B) 12, 13.

The structure of the FAZ has been described in detail using various microscopy techniques but until recently only a few molecular components were known 1, 2, 14. Here we discuss the nature and functions of the FAZ proteins and their assembly in the context of the key roles the FAZ plays in defining and maintaining the shape and form of *T. brucei* and ensuring that this shape is faithfully inherited at division. This linear feature of the trypanosome can be thought of as a ‘cellular ruler’ whose length and constitution facilitates changes in cell form.

## Cell Morphology and FAZ Structure

**Figure 2:**
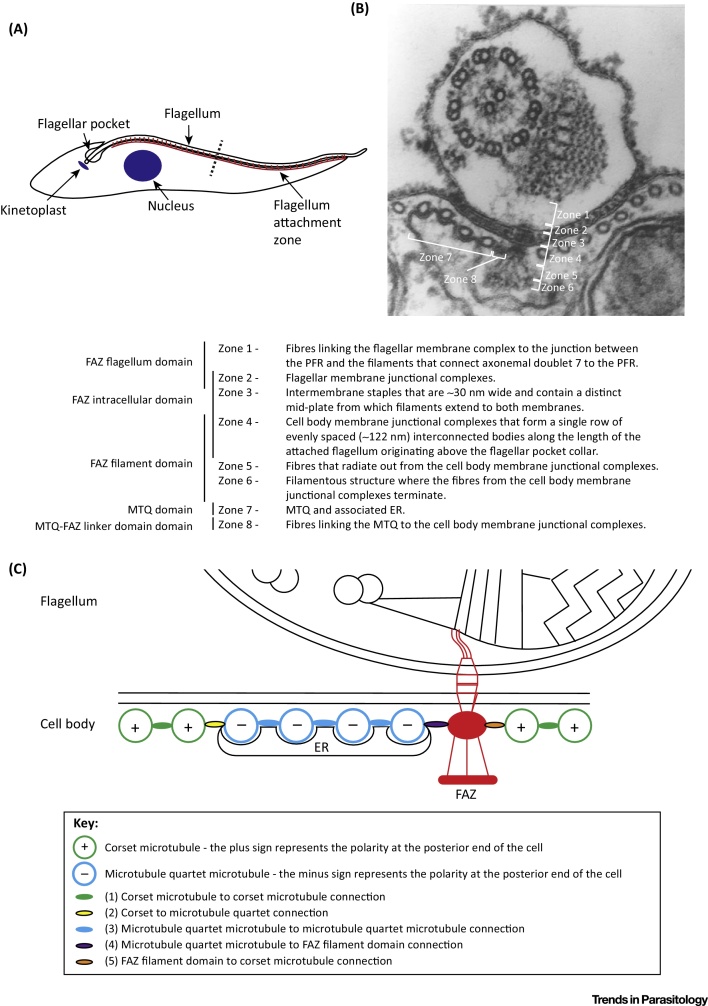
Organisation of the Flagellum Attachment Zone (FAZ) in *Trypanosoma brucei*. (A) Simplified cartoon of the key components of a *T. brucei* trypomastigote cell. Arrows indicate the flagellum, FAZ, flagellar pocket, nucleus, and kinetoplast, with the FAZ highlighted in red and the nucleus and kinetoplast highlighted in blue. The dashed line across the flagellum and FAZ indicates the region where the transverse section in (B) and (C) was taken. (B) Transmission electron microscopy image showing a transverse section of the FAZ with the different zones and structures within them. Abbreviations: ER, endoplasmic reticulum; MTQ, microtubule quartet; PFR, paraflagellar rod. Reproduced from [2]. (C) Cartoon of the transverse section shown in (B) highlighting the asymmetric seam generated in the corset microtubules by the FAZ and MTQ. The five different connections between the various components are shown.

The distinctive shape of a trypomastigote is maintained by a helical, crosslinked, subpellicular array of microtubules arranged in parallel with their plus ends facing towards the posterior end of the cell [15]. The mitochondrial genome (kinetoplast) is linked to the flagellum basal body 16, 17, 18. The flagellum emerges from the cell and is attached to the cell body along most of its length. Flagellum attachment is mediated by the FAZ, which is a large, interconnected set of fibres, filaments, and junctional complexes linking the flagellum skeleton through both the flagellum and cell body membranes to a specialised FAZ filament and associated microtubule quartet (MtQ) 1, 2, 14, 15. The MtQ is antiparallel to the other subpellicular microtubules in the cell body. The FAZ and adjacent MtQ generate an asymmetric seam within the microtubule corset. In transverse sections the organisation of the corset microtubules (polarity: plus end at posterior end of cell) is interrupted by the MtQ (polarity: minus end at posterior end of cell) and the FAZ [15]. Five different linkage types might therefore exist to orchestrate this internal organisation of the FAZ, besides those that link components to the cell membrane (Figure 2). The identification of components of these linkages will be important for our understanding of FAZ biogenesis and function. The definition of trypanosome shape and polarity is therefore derived from the internal cytoskeleton and the flagellum position; this arrangement then coordinates the position of the single-copy organelles 19, 20.

Moving towards a molecular description of the FAZ will require definition of precise protein locations within this complex structure; therefore, in Figure 2 we have defined the major structural zones of the FAZ. Combinations of these zones can now be used to describe different domains of the FAZ, creating a hierarchical descriptive terminology (Figure 2). There is some overlap between the domains but given the present state of knowledge we believe it is useful to have a level of ambiguity.

(i) Zones 1 and 2 constitute the FAZ flagellum domain.
(ii) Zones 2, 3, and 4 constitute the FAZ intracellular domain.
(iii) Zones 4, 5, and 6 constitute the FAZ filament domain.
(iv) Zone 7 constitutes the MtQ domain.
(v) Zone 8 constitutes the MtQ–FAZ linker domain.

There is an invagination of the cell membrane around the base of the flagellum called the flagellar pocket (FP), around which the MtQ wraps helping to define its overall shape. The FP is the only known site for exo- and endocytosis in the trypanosome and is the location of many receptors such as the transferrin receptor 21, 22, 23. The FP forms an interface where signals to/from the host are integrated and processed and hence has a central role in trypanosome pathogenicity. The FAZ also has a potential role in pathogenesis, as the bloodstream form (BSF) in all trypanosomatids examined is a trypomastigote (Figure 1A), and therefore the evolution of extended flagellum attachment appears advantageous in this ecological niche [24]. One possible advantage of flagellum attachment is that it allows host immune system factors bound to the flagellum to be moved towards the FP by hydrodynamic flow or other mechanisms 24, 25.

The overall FAZ structure has similarities to cell–cell adhesive junctions such as desmosomes in multicellular organisms and hence was described in trypanosomes using the term macula adherens [1]. Desmosomes are large, complex cytoskeletal structures spanning two plasma membranes normally connecting intermediate filament bundles in separate adjacent cells [26]; however, autocellular adherens junctions between regions of the same cell like the FAZ are found in certain cells of the trachea of *Drosophila* [27]. Desmosomes comprise two ‘half units’ positioned on adjacent cells; a half unit contains a cytoplasmic plaque that connects intermediate filaments to transmembrane proteins called cadherins that bind to the cadherins of another half unit [28]. The analogy of the desmosome and protein interaction domains within it is potentially useful for informing molecular models for the organisation of the FAZ and flagellum adhesion (FLA) proteins, as discussed below.

## FAZ Protein Location Often Predicts RNAi Phenotype

**Figure 3:**
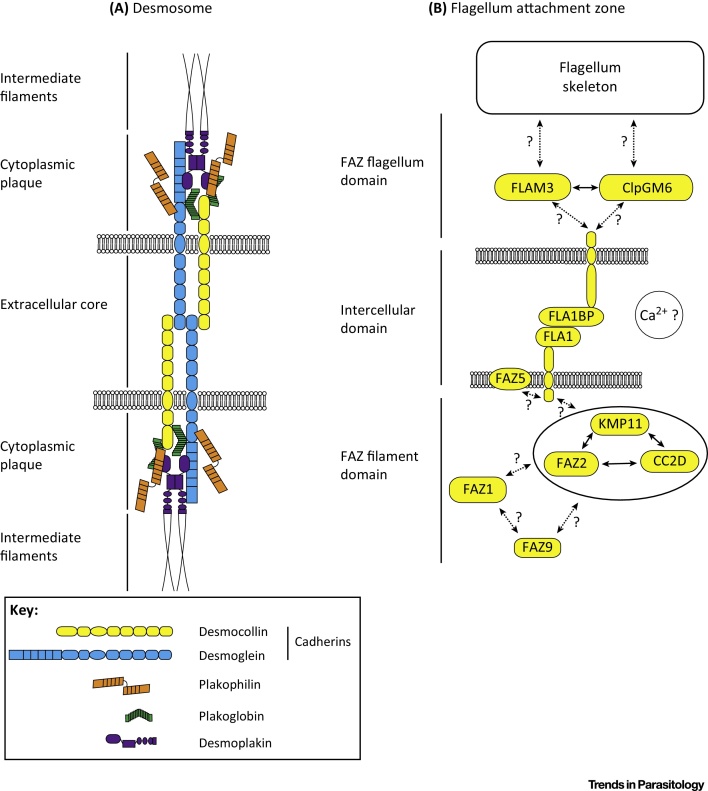
Comparison of the Molecular Organisation of a Desmosome with a Flagellum Attachment Zone (FAZ). (A) Desmosomes are found in multicellular organisms and are large structures that connect together the cytoskeleton of two cells through two plasma membranes; hence, they can be considered analogous to the FAZ. The desmosome model was adapted from [28] and shows the key protein components. The cadherins desmocollin and desmoglein constitute the extracellular core of the desmosome and connect the two halves of the desmosome together. The cadherins are connected into the cytoplasmic plaque by various proteins including plakophilin, plakoglobin, and desmoplakin that in turn bind to the intermediate filaments. (B) Potential interactions and localisations of a selected group of FAZ proteins are shown. Interactions with experimental evidence are shown with a solid line with other possible interactions shown with a dotted line and question mark. Calcium ions are included in the model, as Vickerman noted that *Trypanosoma brucei* harvested from an animal and then grown in blood containing citrate (a calcium chelator) had detached flagella [1]. This is a highly speculative model; however, it will provide a framework on which to integrate new insights into FAZ protein function and helps to highlight the paucity of knowledge about the FAZ compared with the desmosome.

FAZ molecular components were first revealed using monoclonal antibodies and these were then used to identify the first FAZ proteins and their encoding genes 3, 4, 29. Subsequent FAZ proteins have been identified using various techniques (Table 1). An important advance in understanding FAZ protein function will be the positioning of the FAZ proteins in Table 1 into the specific FAZ zones, although we recognise the difficulties. As a start, it is now possible to locate some known FAZ proteins to their respective FAZ domains. We have made an initial attempt to integrate some of these proteins into a model of their potential interactions (Figure 3). This is the first step towards eventually allowing us to understand the interaction details in the same depth as those of the desmosome (Figure 3).

In addition to the FAZ domains’ transverse heterogeneity, there is also heterogeneity in protein localisation longitudinally along the FAZ length 5, 30. We now discuss FAZ proteins and their function and demonstrate that a pattern has emerged linking the RNAi phenotype observed and protein localisation. In general, results so far indicate that if the FAZ protein is located within the FAZ flagellum domain, RNAi leads to shortening of the FAZ and epimastigote-like morphology, whereas if the protein is present in the FAZ intracellular domain RNAi results in flagellum detachment 6, 8, 9, 10. Finally, if the protein is present in the FAZ filament domain RNAi results in either flagellum detachment or kinetoplast or nucleus positioning effects 4, 5, 31.

### FAZ Flagellum Domain (Zones 1 and 2)

We showed that calpain-like protein GM6 (ClpGM6) localised to the FAZ flagellum domain and on depletion of this protein the length of flagellum attachment to the cell body was shortened [9]. The cells underwent a remarkable morphological transition, changing from a trypomastigote to an epimastigote-like configuration with the kinetoplast juxtaposed or anterior to the nucleus with a much shorter FAZ. There was also a coordinate relocation of associated organelles such as the Golgi and FP. Remarkably, ClpGM6 RNAi was not lethal and cells with the epimastigote-like morphology continued to proliferate in culture; however, the ability of this cell line to infect tsetse flies has not been assessed. The phenotype resulting from the depletion of FLAgellar Member 3 (FLAM3), another FAZ flagellum domain protein recently examined 7, 10, showed similarity to ClpGM6; depletion of FLAM3 resulted in the cells transitioning from a trypomastigote to an epimastigote-like morphology [10]. We defined a dependency of expression between FLAM3 and ClpGM6, with loss of one resulting in the reduction of expression of the other [10]. It is possible that ClpGM6 and FLAM3 are part of the same complex, suggesting that individual FAZ proteins group into large-scale molecular complexes and facilitating an understanding of the FAZ assembly hierarchy.

During the *T. brucei* life cycle, trypanosomes adopt different morphologies with different FAZ lengths (Figure 1B). Rotureau and colleagues [13] examined the FAZ during the life cycle and showed that there was a drop in labelling with monoclonal antibodies specific to the FAZ between mesocyclic and epimastigote cells coinciding with a switch from trypomastigote to epimastigote morphology. In trypanosomes, protein expression regulation occurs at the post-transcriptional level and the RNA-binding proteins ALBA3/4 have been implicated in the transition to the epimastigote form [32]. On knock down of ALBA3/4, cells begin to adopt an epimastigote morphology and RNA-seq analysis of these cells showed that the ClpGM6 transcript was the one most highly downregulated [32]. The FAZ has previously been implicated in nuclear positioning during cell division [4]; does this drop in FAZ protein expression allow the migration of the nucleus that occurs between the mesocyclic and epimastigote forms?

### FAZ Intracellular Domain (Zones 2, 3, and 4)

FLA1 was identified in *T. brucei* due to its similarity to the FAZ protein GP72 from *Trypanosoma cruzi* [29]*.* Interestingly, GP72 was identified by an anticarbohydrate monoclonal antibody, WIC29.26, and subsequent publications have revealed that the glycoprotein has an unusual sugar composition 33, 34, 35, 36, 37. Several studies revealed the involvement of specific carbohydrates and glycoproteins in flagellum attachment, including one publication that showed that loss of GDP-fucose production resulted in flagellum detachment 6, 29, 38, 39, 40, 41, 42, 43. However, overall the role of specific carbohydrates has not been much explored; this seems odd given the significance of the early finding, as the presence of glycoconjugates adds another layer of complexity and another potential regulatory step to the FAZ structure.

Both FLA1 and GP72 are transmembrane proteins with a small intracellular domain and a glycosylated extracellular region containing NHL repeats, which is a widespread protein interaction domain [44]. Immunoprecipitation of FLA1 identified FLA1-binding protein (FLA1BP), which is predicted to be a glycosylated transmembrane protein with a large extracellular region containing NHL repeats and a small intracellular domain 8, 45. Using a combination of tagged FLA1 and FLA1BP and RNAi, Sun and colleagues demonstrated that FLA1 is located on the cell body membrane and FLA1BP on the flagellum membrane [8]. Given that these proteins interact, we suggest they may be functionally analogous to the desmosomal cadherins by connecting the FAZ flagellum and FAZ filament domains (Figure 3).

The interaction between FLA1 and FLA1BP was shown in procyclic forms (PCFs) but the expression of these proteins is downregulated in BSFs [46]. The BSF surface coat (variant surface glycoprotein) is structurally distinct from the PCF surface (procyclins), suggesting a reason why BSF equivalents of FLA1 and FLA1BP have evolved. *T. brucei* contains two proteins (FLA2 and FLA3) that have ∼60% identity to FLA1 with the same domain structure and are upregulated in BSFs 6, 8, and therefore are likely to be able to substitute for FLA1. In addition, another BSF-specific protein that localises to the FAZ was identified encoded by near-identical genes (Tb927.5.4570 and Tb927.5.4780) that has ∼40% identity to FLA1BP with the same domain structure; unfortunately, this was also named FLA3 43, 45. Currently there is no evidence of interaction between FLA3 43, 45 and FLA2/3 6, 8 proteins and so this confusing nomenclature will best be refined when more is known about the biochemistry of these proteins.

FLA1 depletion led to flagellum detachment and eventually cell death, showing that FLA1 is required for flagellum attachment [6]. Interestingly, the *T. cruzi* knockout cell line of the FLA1 orthologue GP72 was viable despite having detached flagella 38, 39. The expression of the *T. cruzi* GP72 protein in *T. brucei* led to flagellum detachment and the cells were viable, albeit with a much reduced growth rate. The sequence divergence between FLA1 and GP72 appears to have resulted in incompatibilities between the binding interfaces required to ensure flagellum attachment, providing a potential route to dissect the function of the different domains of FLA1 [6]. As with FLA1 RNAi, FLA1BP knock down resulted in full-length detachment of the flagellum and also a reduction in both FAZ length and cell body length, resulting in the production of cells with an epimastigote-like morphology. The appearance of cells with an epimastigote-like morphology fits with the RNAi phenotype pattern described above, as FLA1BP is located within the FAZ flagellum domain in addition to being in the FAZ intracellular domain. However, unusually for a flagellum detachment phenotype, the cells continued to proliferate [8]. In the literature there is no clear correlation between growth and the amount of flagellum detachment. Our experience suggests that how cells are handled and prepared for microscopy has a major effect on the apparent level of detachment. It will be of interest to make quantitative comparisons over long periods of culture with careful handling to ensure that the images of the cells on the slide are representative of the live cells in the culture flask. Experience shows that, in some protocols, during the washing and settling of the cells onto a slide the flagellum tends to become detached if the FAZ structure has been weakened; therefore, the speed and duration of the centrifugation washing steps, resuspension protocols, and the slide adherence method all have an effect. Comparative studies of different mutant phenotypes using one protocol will be useful in the future, as will careful comparison of live cells or cells fixed directly in culture with those imaged as fully processed, labelled cells or cytoskeletons.

### FAZ Filament Domain (Zones 4, 5, and 6)

Both coiled-coil C2 domain-containing protein (CC2D) and FAZ2 localise to the FAZ filament domain and depletion of either leads to inhibition of FAZ assembly causing full-length flagellum detachment and cell death 5, 30, 47. The reduction in FAZ length led to a decrease in cell body length during the knock down of both CC2D and FAZ2. A FAZ1 stub was still able to form near the FP in these knock down cells, suggesting that FAZ nucleation can occur without a full complement of FAZ proteins 5, 47.

FAZ1 is also found in the FAZ filament domain; however, the phenotype observed during FAZ1 knock down was different. There was no full-length flagellum detachment; instead, there were flagellum attachment defects. There was also no change in cell body length, as there was no effect on overall FAZ length [4]. However, the FAZ architecture was disorganised, with changes in the labelling patterns of anti-FAZ antibodies [4].

The function of FAZ9, a FAZ filament domain protein, has been analysed by RNAi 30, 31. On FAZ9 depletion the flagellum remained attached to the cell body; however, the kinetoplast was positioned anterior to the nucleus. This is similar to the ClpGM6 and FLAM3 RNAi phenotypes except that there was no change in the overall length of the FAZ on FAZ9 RNAi 9, 10, 31. The phenotypes observed for the knock down of these FAZ filament domain proteins may be dependent on the zone in which the proteins are found. FAZ9 may be located more towards zone 6, hence affecting kinetoplast and nucleus positioning (Figure 2), whereas CC2D and FAZ2 may be in zone 4, resulting in flagellum detachment with FAZ1 in zone 5, where knock down has a less dramatic effect on flagellum attachment.

Zoids are anucleate cytoplasts that are commonly observed in FAZ protein RNAi phenotypes 4, 5. They have a kinetoplast, basal body, and flagellum, suggesting that the positioning of the cytokinesis furrow facilitates inclusion of such organelles via the distal end of the FAZ marking the site of cytokinesis ingression [15]. Also, a clear correlation between FAZ length and cell body length exists and hence coordination between FAZ assembly and the subpellicular microtubule assembly 5, 8, 47. Overall, these data on the function of the FAZ and its constituent proteins demonstrate that the FAZ acts as a cellular ruler for trypanosome morphology, with changes in FAZ protein composition determining both FAZ length and cell body size and also organelle layout.

Besides the dynamic nature of the FAZ as a cellular ruler of cell architecture, it is also likely that it operates to provide a physical platform for spatial biochemistry enabling the specific localisation of regulatory components like polo-like kinase 31, 48, 49, 50, 51.

## FAZ Proximal-End Assembly and Implications

**Figure 4:**
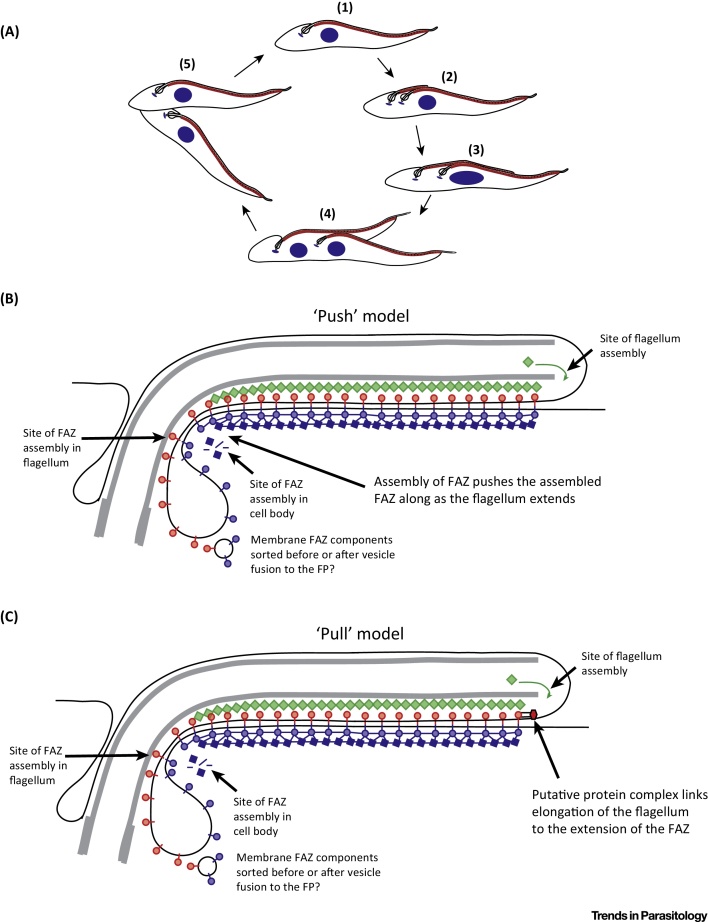
The Flagellum Attachment Zone (FAZ) Assembly ‘Pull’ Model. (A) A simplified schematic of the *Trypanosoma brucei* cell cycle. The cell begins to assemble a new flagellum and FAZ (highlighted in red) alongside the old one, followed by kinetoplast division (2). The new flagellum continues to extend alongside the old flagellum until the ‘stop point’ is reached after which the new flagellum does not extend along the old flagellum any further (3). After the stop point is reached the two kinetoplasts separate further, followed by mitosis (4) and finally cytokinesis (5). (B) The FAZ assembly ‘push’ model with the assembling FAZ pushing out the assembled FAZ as the flagellum elongates. FAZ proteins are transported to the proximal site of assembly; however, the exact mechanisms that target these components remain to be elucidated. (C) The FAZ assembly ‘pull’ model with a putative protein complex in the flagellum ‘pulling’ out the assembling FAZ.

We have elucidated the site of FAZ assembly, using cells where the expression of FAZ proteins present in each of the three domains of the FAZ was under the control of an inducible promoter [30]. For every protein examined, the newly synthesised FAZ proteins localised to the proximal end of the FAZ (the FP end), which is at the opposite end to the addition site for flagellar components, showing that the overall flagellum/FAZ structure has two distinct assembly sites (Figure 4A). Others have subsequently confirmed this result and reiterated our original model [47].

Our FAZ proximal addition assembly model was confirmed by examining RNAi knock down phenotypes. The model predicts that in a cell where the construction of a new FAZ had begun before initiation of FAZ protein knock down, the distal end of the FAZ structure would be stably assembled; however, the proximal end would be depleted in the targeted FAZ protein. After short RNAi inductions of FLA1 or FAZ5 RNAi, a loop of detached flagellum was observed at the proximal end near the FP, demonstrating that the distal end of the FAZ was stable and the proximal end unstable [30].

The opposite-polarity assembly of the FAZ and flagellum has many implications for the construction of this complex cytoskeletal interface. We focus on:

(i) coordinating assembly between the flagellum and FAZ;
(ii) FAZ protein targeting;
(iii) FAZ and flagellum connections;
(iv) hierarchy and temporal order of assembly;
(v) maintenance of attachment.

### Coordination

The addition of LiCl has been shown to lengthen the flagellum in several organisms including trypanosomes and is therefore a useful method for manipulating the flagellum and the FAZ structure. In the presence of LiCl, trypanosomes assembled both a longer flagellum and a longer FAZ [8]. However, disruption of intraflagellum transport (IFT) by RNAi resulted in cells that were unable to assemble a flagellum yet maintained their old flagellum [52]. There was a clear FAZ1 signal associated with the expected position of the new flagellum, although the length of the FAZ was much shorter [52]. Moreover, IFT RNAi also resulted in the generation of a flagellar sleeve – a thin tube of membrane pulled out by the flagella connector – and this sleeve was laterally attached to the cell body via a FAZ [53]. Together these findings suggest that there is coordination between flagellum and FAZ extension and that initial nucleation and extension of the FAZ can occur without an assembling flagellum skeleton but for full-length FAZ assembly an intact flagellum structure is required.

How is flagellum assembly linked to that of the FAZ? As addition of FAZ proteins to the assembling FAZ occurs at the proximal end, two simple models can be envisaged for the forces that coordinate the elongation of the FAZ with the flagellum. First, FAZ subunit addition at the proximal end could ‘push’ the FAZ along as the flagellum extends (Figure 4A,B). Second, a structure associated with the assembling end of the flagellum and connected to the distal end of the FAZ could ‘pull’ the FAZ, allowing new subunits to be added at the proximal end (Figure 4C). In *T. brucei* the new flagellum extends alongside the old flagellum until it reaches the stop point and then does not extend any further along the old flagellum [53]. It should be noted that the pull force is required only until the stop point has been reached and after this the FAZ remains fixed in position relative to the cell body and the extension of the cell posterior allows the proximal-end assembly of the FAZ to continue (Figure 4A).

### Targeting

The FAZ structure is complex, with proteins present in the flagellum, cell body, membranes, and intermembrane spaces, and thus will require targeting or retention mechanisms to ensure correct localisation; it is likely that the components located in the flagellum will have targeting or retention motifs different to those in the cell body.

Moreover, there are proteins associated with the membrane of either the flagellum or the cell body, which will be trafficked through the FP. However, the stage at which these proteins are sorted is not known: does it occur before fusion to the FP, giving rise to two different vesicle populations that then fuse in distinct regions of the FP, or does the sorting occur within the FP? Currently, no targeting signal for any FAZ protein has been fully delineated; however, the short cytoplasmic domain of both FLA1 and FLA1BP was shown to play a role in their localisation to the FAZ [8].

Further to this, how and where is the intracellular domain formed that connects the flagellum and cell body membranes, given that proteins present in zones 2 and 4 will be trafficked differently either to the flagellum or to the cell body? Do the proteins on opposing membranes snap together in the neck region of the FP while being assembled into the FAZ structure?

We suggest that FAZ substructures may be preassembled and then transported to the FP region where they are integrated into the FAZ but accumulate if FAZ or flagellum assembly is disrupted. This would be analogous to desmosome assembly, where preassembled desmosomal complexes are stored in vesicles and then transported to the cell surface when required [54]. Perhaps an accumulation of electron-dense structures at the base of the flagellum in the *T. cruzi GP72* knockout cell line represents evidence of this [55].

### Connections

The FAZ and flagellum most likely slide over each other as they are assembled and so understanding when the connections between the two structures are made and when they are consolidated is important. The sliding is potentially comparable with the actin/myosin sliding filament model in muscle where overall connectivity is maintained but individual connections are made and broken consecutively. Initially weak connections may be established that are easily broken as the two structures slide over each other, being consolidated only once the structure is mature. Increasing FLAM3 content may be an indication of the strengthening and maturing of the FAZ–flagellum connections [10].

### Hierarchy

We know little about the hierarchy and temporal order of FAZ assembly. RNAi depletion of an intracellular domain protein (FAZ5) for a short period of time resulted in detachment of the new flagellum at its proximal end and a FAZ flagellum domain protein (ClpGM6) signal was lost from the detached portion of the flagellum; however, a cell body FAZ protein (FAZ1) signal remained present below the detached section [30]. This suggests that the flagellum FAZ proteins require the membrane FAZ proteins for assembly and that filament assembly is independent of flagellum and membrane FAZ components. In addition we have demonstrated the interdependency of FLAM3/ClpGM6 expression and localisation [10].

### Maintenance

The mature flagellum and FAZ must exhibit strong connections between the structures to ensure that the flagellum remains attached despite beating. Flagellum detachment and attachment errors were observed on the knock down of certain flagellar proteins such as calmodulin, KIF9B, and PF16. Depletion of calmodulin or KIF9B results in severe defects in the assembly of an extra-axonemal structure called the paraflagellar rod (PFR), whereas loss of PF16 results in aberrant orientation of the central-pair microtubules 56, 57, 58. KIF9B depletion causes loops of flagellum to become detached, whereas PF16 or calmodulin knock down results in full-length flagellum detachment. KIF9B depletion did not affect FLA1 or FLAM3 addition [7], demonstrating that FAZ assembly had occurred. Cells with detached flagella after calmodulin knock down showed a distinct line of blobs along an indent in the cell surface on scanning electron microscopy. These blobs appear to be flagellum attachment structures, suggesting that the flagellum was initially attached but then became detached [56]. Overall these data suggest that the maintenance of flagellum attachment requires an intact axoneme or PFR skeleton but these structures are not required for FAZ assembly.

## FAZ in Other Kinetoplastids

Many kinetoplastids have an attached flagellum and all FAZ proteins identified so far are kinetoplastid specific. FAZ function has mainly been studied using trypanosomes, so the FAZ function in species such as *Leishmania* that exist as either promastigotes or amastigotes is rather more enigmatic. It is possible that the FAZ functions, as in *T. brucei*, to define flagellum axial positioning and cytokinesis events [15]. The kinetoplastids are able to adopt a wide variety of different shapes; however, the genomes of these organisms have well-maintained chromosomal synteny and sequence conservation between genes 59, 60, 61. We have shown that the morphological changes that occur during ClpGM6 RNAi indicate that relatively small changes in gene expression can cause major changes in cell shape [9]. Therefore, different trypanosome morphologies are likely to be assembled using the same basic set of building blocks [9].

## Concluding Remarks

Here we have argued that the FAZ acts as a cellular ruler for trypanosome morphology and is crucial in determining the overall length of the cell and the positioning of the organelles within it. Furthermore, it is likely that the wide range of kinetoplastid morphologies can be achieved through small modulations in expression of a conserved FAZ gene set rather than requiring a different gene set for each specific morphology. This conclusion has implications for understanding kinetoplastid evolution and cell-type gene expression.

An ever-expanding number of FAZ proteins are being revealed; however, currently this is little more than a list. Looking forwards we need to define subcomplexes and the assembly interdependencies of complexes; this will be greatly aided by localising these proteins into the FAZ zone system defined here and elucidating their targeting or retention mechanisms, and we have highlighted these issues in the Outstanding Questions. This area of trypanosome cell biology is at last providing much-needed insight into how these parasites acquire and morph cell shape and form.

Outstanding Questions Where are the known FAZ proteins specifically located within the FAZ zonal structure?

What are the targeting signals that ensure the FAZ proteins are correctly localised?

Which FAZ proteins interact with each other and in what temporal order to orchestrate assembly?

What is the function of the FAZ in kinetoplastid cell types with little flagellum attachment?

How are the FAZ assembly processes coordinated within two different cellular compartments?

## Figures and Tables

**Table 1:** Known FAZ Proteins

Gene ID	Name	Localisation	Localisation Method	Discovery Route	Molecular Weight (kDa)	Known Domains	Coiled Coils	Repeats	BSF or PCF Expression	Refs
Tb927.8.4010	FLA1	Intracellular domain	Immunofluorescence	Monoclonal antibodies	59	NHL domain	No	No	PCF	[29]
Tb927.8.4060	FLA2	Intracellular domain	Not done	Bioinformatic	64	NHL domain	No	No	BSF	[6]
Tb927.8.4110	FLA3	Intracellular domain	Not done	Bioinformatic	64	NHL domain	No	No	BSF	[8]
Tb927.8.4050	FLA1BP	Intracellular domain	eYFP tagging and immunofluorescence	Immunoprecipitation	83	NHL domain	No	No	PCF	[8]
Tb927.8.4100	FLA1BP	Intracellular domain	eYFP tagging and immunofluorescence	Immunoprecipitation	83	NHL domain	No	No	PCF	[8]
Tb927.5.4570	FLA3	Intracellular domain	Immunofluorescence	Lectin binding	89	NHL domain	No	No	BSF	[43]
Tb927.5.4580	FLA3	Intracellular domain	Immunofluorescence	Lectin binding	89	NHL domain	No	No	BSF	[43]
Tb927.10.2880	Ca^2+^ channel	Intracellular domain	Immunofluorescence	Proteome validation	304	Ion transporter	Yes	No	Both	[62]
Tb927.10.8830	FAZ5	Intracellular domain	eYFP tagging	Immunoprecipitation	67	Chaperone J domain	Yes	No	Both	[30]
Tb927.10.14320	FAZ9	FAZ filament domain	eYFP tagging	Bioinformatic	122	ARM repeats	Yes	No	Both	[30]
Tb927.11.1090	ClpGM6	FAZ flagellum domain	Immunofluorescence	RNAi screen	>660	Calpain	Yes	Yes	Both	[9]
Tb927.8.4780	FLAM3	FAZ flagellum domain	eYFP tagging	Proteome validation	468	Clu domain	Yes	Yes	Both	[13]
Tb927.1.4310	FAZ2	FAZ filament domain	eYFP tagging	Immunoprecipitation	184	No domains	Yes	Yes	Both	[30]
Tb927.11.12530	FAZ3	FAZ filament domain	eYFP tagging	Immunoprecipitation	91	No domains	Yes	No	Both	[30]
Tb927.7.3330	FAZ10	Unknown	Immunofluorescence	BioID	503	PFAMB506	Yes	Yes	Both	[63]
Tb927.4.2060	FAZ8	FAZ filament domain	eYFP tagging	Bioinformatic	67	PFAMB506	Yes	No	Both	[30]
Tb927.4.3740	FAZ1	FAZ filament domain	Immunofluorescence and immunoelectron microscopy	Monoclonal antibodies	193	PFAMB506	Yes	Yes	Both	[4]
Tb927.4.2080	CC2D	FAZ filament domain	Immunofluorescence and eYFP tagging	Proteome validation	105	C2 domain/PFAMB14968	Yes	No	Both	[5]
Tb927.9.10530	FAZ4	FAZ filament domain	eYFP tagging	Immunoprecipitation	119	PFAMB506	Yes	No	Both	[30]
Tb927.10.840	FAZ6	FAZ filament domain	eYFP tagging	Immunoprecipitation	195	WD domain/PFAMB506	Yes	No	Both	[30]
Tb927.4.5340	FAZ11	Unknown	Immunofluorescence	BioID	95	No domains	Yes	No	Both	[63]
Tb927.10.15390	FAZ7	FAZ filament domain	eYFP tagging	Immunoprecipitation	124	Kinesin	Yes	No	Both	[30]
Tb927.11.13230	TbVAP	FAZ filament domain (ER)	Immunofluorescence	Proteome validation	24	MSP domain	No	No	Both	[20]
Tb927.9.13820	KMP11	Cell body and flagellum	Immunofluorescence	Immunoprecipitation	11	KMP11 domain	Yes	No	Both	[47]
Tb927.9.13880	KMP11	Cell body and flagellum	Immunofluorescence	Immunoprecipitation	11	KMP11 domain	Yes	No	Both	[47]
Tb927.9.13920	KMP11	Cell body and flagellum	Immunofluorescence	Immunoprecipitation	11	KMP11 domain	Yes	No	Both	[47]
Tb927.11.2590	FAZ12	FAZ filament domain	Immunofluorescence	BioID	121	No domains	Yes	No	Both	[48]
Tb927.3.1020	FAZ13	FAZ filament domain	Immunofluorescence	BioID	54	No domains	Yes	No	Both	[48]
Tb927.8.6980	FAZ14	FAZ filament domain	Immunofluorescence	BioID	95	no domains	Yes	No	Both	[48]
Tb927.11.3300	TbSAS4	Anterior tip of FAZ filament domain	Immunofluorescence	BioID, bioinformatics	108	T-complex protein 10 C terminus	Yes	No	Both	31, 48
Tb927.8.7070	FAZ15	Unknown	Immunofluorescence	Phosphoproteomics	35	No domains	No	No	Both	[31]
Tb927.5.3460	FAZ16	Unknown	Immunofluorescence	Phosphoproteomics	57	LysM domain	No	No	Both	[31]
Tb927.10.7210	FAZ17	Unknown	Immunofluorescence	Phosphoproteomics	26	No domains	No	No	Both	[31]
Tb927.11.15800	TOEFAZ1	Anterior tip of FAZ filament domain	Immunofluorescence	BioID, phosphoproteomics	90	No domains	Yes	No	Both	[31]
